# 

**Published:** 1910-09-15

**Authors:** 


### ANNOUNCEMENTS.

Oral Surgery. Dr. Truman W. Brophy is writing a book on Oral Surgery which will be published this fall by P. Blakeston's Son & Co. Dr. Brophy has the technical skill and experience of many years of teaching this subject, and we know of no one who should write a more acceptable book on the subject. We shall await its appearance with great pleasure.

---

ANATOMY. Lea and Febiger, have issued a new edition of Gray's Anatomy which has been thoroughly revised by Dr. E. A. Spitzka. Dr. Spitzka is professor of Anatomy in Jefferson Medical College, and is recognized as an eminent authority and teacher. The work of revision could not be entrusted to better hands. If he succeeds in improving Gray's Anatomy he will accomplish a most creditable task, as this work has been standard for so many years that we cant think it capable of serious revision. This is the 19th edition. Professor Spitzka has undoubtedly improved the illustrations as he is an expert artist and investigator. Considerable old matter has been replaced to bring it more in harmony with present practice of surgery. While there are fewer pages, the work is the most comprehensive text book on Anatomy published. It is more than a reprint; it is a real revision and both Editor and publishers are to be congratulated on the scientific and artistic result.

---

NEW YORK ALUMNI ASSOCIATION, XI PSI PHI FRATERNITY. The annual fall meeting and election of officers of the New York Alumni Association, Xi Psi Phi Fraternity will be held at Healy's Columbus Avenue, and 66th street, New York City, at 8 P. M., Wednesday October 12th., 1910. Every XI PSI PHI ALUMNUS residing in or about New York City is urged and expected to be present.

